# Sickened by the Weather: Exploring the Climatic Impact on West Nile Virus (WNV) and *Legionella pneumophila* in Piedmont—A Retrospective Observational Study (2021–2024)

**DOI:** 10.3390/idr18010018

**Published:** 2026-02-12

**Authors:** Paolo Valesella, Antonio Curtoni, Alessio Leone, Marco Iannaccone, Fabrizia Pittaluga, Elisa Zanotto, Alessandro Bondi, Rocco Francesco Rinaldo, Nour Shbaklo, Silvia Corcione, Simone Baldovino, Irene Cecchi, Elisa Menegatti, Paolo Solidoro, Cristina Costa

**Affiliations:** 1 Microbiology and Virology Unit, AOU Città della Salute e della Scienza di Torino, 10126 Turin, Italy; pvalesella@cittadellasalute.to.it (P.V.); fpittaluga@cittadellasalute.to.it (F.P.); alessandro.bondi@unito.it (A.B.);; 2 Department of Public Health and Paediatrics, University of Turin, 10124 Turin, Italy; 3 Division of Respiratory Medicine, Cardiovascular and Thoracic Department, AOU Città della Salute e della Scienza di Torino, 10126 Turin, Italy; 4 Department of Medical Sciences, University of Turin, 10124 Turin, Italy; 5 School of Medicine, Tufts University, Boston, MA 02111, USA; 6 Department of Clinical Pathology and Clinical Biochemistry, University of Turin, 10124 Turin, Italyelisa.menegatti@unito.it (E.M.)

**Keywords:** climate change, global health, West Nile Virus, *Legionella pneumophila*, global warming, vector-borne disease, emerging pathogens

## Abstract

Background: Climate change represents a major global health challenge, with rising temperatures and altered precipitation patterns influencing the spread of infectious diseases. This study investigated the association between climatic factors (average temperature and precipitation) and the monthly proportion of laboratory-confirmed *Legionella pneumophila* serogroup 1 and West Nile Virus infections among clinically suspected patients in a large teaching hospital in Northern Italy. Methods: We retrospectively analyzed data from 2021 to 2024. The primary outcome was the monthly proportion of positive tests (standardized per 1000 clinically suspected patients) for *Legionella pneumophila* serogroup 1 (urinary antigen) and West Nile Virus (serology). Associations with climatic variables were assessed using linear and multivariate regression models, as well as Generalized Additive Models (GAMs). Seasonal effects were evaluated through ANOVA. Results: For *Legionella pneumophila*, precipitation was not significantly associated with the proportion of positive tests (*p* = 0.1438; R^2^ = 0.049). In contrast, average temperature was a significant predictor: each 1 °C increase was associated with +0.52 positive cases per 1000 tested patients (*p* = 0.000283; R^2^ = 0.267). Multivariate models confirmed temperature as the dominant factor. For West Nile Virus, precipitation showed no meaningful effect (*p* = 0.914). However, average temperature demonstrated a significant positive association with the proportion of positive cases (*p* = 0.00293; coefficient = 9.33), with seasonal analysis highlighting a marked summer peak (mean = 399.68 positive cases per 1000 tested; *p* = 0.00653). Conclusions: Our findings underline the predominant role of temperature over precipitation in driving the burden of both *Legionella pneumophila* and West Nile Virus infections among hospitalized patients. These results strengthen the evidence that the life cycles of these pathogens are tightly climate-dependent. Developing effective adaptation strategies is essential to mitigate climate-related health risks.

## 1. Introduction

Climate change stands as one of the most formidable challenges to global health in our era, threatening fundamental aspects of human existence such as clean air, safe drinking water, and food security [1]. The Intergovernmental Panel on Climate Change (IPCC) has emphasized that human-induced climate change poses significant risks to public health worldwide [1,2]. The repercussions of climate change, including extreme weather events and indirect effects such as the spread of food, water, and vector-borne diseases, undermine decades of progress in social, technological, and healthcare sectors [1,2,3]. This is particularly concerning since the life cycles of numerous infectious agents depend on climatic conditions [4,5].

Extensive research has shown that variations in temperature and precipitation significantly influence the distribution and transmission dynamics of infectious diseases. The geographical expansion of mosquitoes and other vectors has been linked to an increase in diseases such as West Nile Virus (WNV), posing heightened risks especially in tropical and subtropical regions [6]. Likewise, changes in hydrological cycles and average temperatures, coupled with phenomena such as desertification and rising sea levels, compromise water and food security and contribute to the spread of waterborne infections such as Legionnaires’ disease [7].

In recent years, the role of climate change in the spread of infectious diseases has been extensively investigated. A brief PubMed search conducted in February 2025 using the keywords “climate change infectious diseases” retrieved 2799 relevant studies published since 1980. Strikingly, 2374 of these were published in just the last decade (2015–2025), with a peak of 398 studies in 2024 alone. Narrowing the focus further, 584 of these studies specifically addressed the impact of temperature changes, while 48 examined the role of precipitation patterns. These findings highlight the scientific community’s growing attention to the influence of climatic variables on infectious diseases.

A closer analysis of studies published between 2015 and 2025 provides further insights into the association between climate change and infectious diseases. For waterborne diseases, 28 reviews and nine original studies reported significant links with climatic factors, most frequently highlighting correlations with precipitation (four studies) and temperature (two studies). Within this category, *Legionella pneumophila* was specifically addressed in three reviews, although without detailed specification of individual climatic parameters [8,9,10].

Vector-borne diseases were even more extensively investigated, with 91 reviews and 49 studies documenting associations, particularly with rising temperatures (47 studies) and precipitation patterns (20 studies). In this group, WNV stood out: 23 reviews and one original study emphasized its sensitivity to climate drivers, reporting positive correlations with both precipitation (four studies) and temperature (12 studies). Interestingly, some studies noted increased incidence during autumn and winter seasons [11,12], while others highlighted divergent findings, for example, one study questioned whether precipitation plays any meaningful role in influencing disease incidence [13].

Taken together, these findings reflect the increasing scientific recognition of the impact of climate variability on both waterborne and vector-borne diseases [14].

*Legionella pneumophila* is a Gram-negative, aerobic, intracellular bacterium belonging to the Legionellaceae family [15]. Among its serogroups, *Legionella pneumophila* serogroup 1 is the most clinically relevant, being responsible for the majority of human infections, including the severe pneumonia known as Legionnaires’ disease [15]. *Legionella pneumophila* thrives in aquatic environments, both natural and artificial. It grows optimally at 37 °C, within a broader range of 25–45 °C. High humidity and stagnant water further favor its proliferation, making water systems such as cooling towers, distribution networks, air conditioning units, and thermal pools ideal habitats [7]. Warm and humid weather, particularly during summer and damp autumn months, significantly increases the likelihood of *Legionella pneumophila* growth, thereby elevating the risk of exposure and infection in enclosed environments [8]. Although relative humidity may represent an additional relevant climatic driver, its specific contribution could not be assessed in the present study because homogeneous monthly data were not available. However, the contribution of individual climatic factors may vary according to data availability and spatial resolution.

In the Piedmont region, legionellosis represents a relevant and growing public health concern. Over the last decade (2014–2023), both in Piedmont and at the national level, a progressive increase in the number of reported legionellosis cases has been observed. This trend was particularly marked in 2023, when 326 cases were recorded in Piedmont, corresponding to an incidence rate of 7.7 cases per 100,000 inhabitants—the highest value ever reported by the regional surveillance system [16].

WNV, first isolated in 1937 in Uganda’s West Nile district, is an arbovirus belonging to the genus Flavivirus within the family Flaviviridae. Today, WNV is distributed worldwide. The virus shows a marked affinity for neuronal cells, a key factor underlying the severe neurological complications that may occur in infected patients. WNV is transmitted primarily by mosquitoes of the Culex genus, which act as vectors between birds and other animals, including mammals and humans [17].

In the Piedmont region, WNV represents an established and clinically relevant arboviral infection. In 2024, compared with the two previous years, the number of reported WNV cases slightly declined but remained substantial, with 21 cases notified (19 confirmed and 2 probable). All cases were classified as autochthonous, with infection acquired within the Piedmont region. In 2023, a total of 58 cases were reported, reflecting a steadily increasing trend from 2021 to 2023, followed by a modest decrease in 2024 [18].

Building on these premises, our study investigated seasonal trends, diagnostic findings, and climatic correlations observed in clinical cases recorded between January 2021 and September 2024 at the Teaching Hospital A.O.U. Città della Salute e della Scienza of Turin (Piedmont, Italy). While most available evidence is based on population-level surveillance data and time-series approaches, fewer studies have explored real-life diagnostic data at the hospital level. We therefore focused on a hospital-based setting and hypothesized that seasonal and climatic variations, particularly temperature and rainfall, were associated with changes in the monthly proportion of laboratory-confirmed *Legionella pneumophila* and WNV infections among clinically suspected patients in our institution.

These pathogens were selected as the focus of our study because they are subject to systematic and standardized diagnostic protocols in our institution. This allowed for the collection of high-quality, continuous data, which is essential for investigating correlations with climatic variables over a multi-year period. By contrast, other climate-sensitive pathogens (such as Aedes-borne arboviruses or enteric bacteria) were excluded because the lack of systematic, year-round diagnostic screening resulted in discontinuous data, which would have precluded meaningful temporal analyses.

## 2. Materials and Methods

A real-life retrospective single-center observational study was designed to evaluate the relationship between climatic variables and the monthly proportion of laboratory-confirmed *Legionella pneumophila* and WNV infections among clinically suspected patients during the period from January 2021 to September 20242024 (Table S1). Microbiological data were retrospectively extracted from the Labora-tory Information System (LIS) of the Microbiology and Virology Unit of the A.O.U. Città della Salute e della Scienza of Turin (Turin, Italy) (Table S2). Environmental data, including average monthly temperature and cumulative monthly precipitation, were obtained from the publicly available monthly climate bulletins of ARPA Piemonte (Regional Environmental Protection Agency, Torino, Italy).

The study population included adult patients tested during the study period for *Legionella pneumophila* serogroup 1 urinary antigen or for WNV IgG and IgM antibodies due to clinical suspicion of infection. Only patients who were hospitalized within 3–4 days after presentation to the emergency department were included, in order to rule out hospital-acquired infections. Repeated tests performed on the same patient were excluded; when multiple tests were available, only the first positive result was considered for analysis.

A positive urinary antigen test for *Legionella pneumophila* serogroup 1 and positivity for WNV IgG and/or IgM serology were considered indicative of *Legionella pneumophila* and WNV infection, respectively.

For *Legionella pneumophila*, urine samples collected in sterile containers were analyzed within 24 h using the BinaxNOW Legionella immunochromatographic assay (Abbott, Abbott Park, IL, USA), with results available within 15 min. Test positivity was assessed either visually by an expert clinical microbiologist or digitally using the DIGIVAL (Abbott, Abbott Park, IL, USA) reader. All positive results were confirmed by heat inactivation at 90 °C, centrifugation, and repeat testing.

For WNV, serum samples were collected and analyzed for IgG and IgM antibodies using an indirect chemiluminescence immunoassay (VIRCLIA^®^ IgG/IgM, Vircell, Granada, Spain). All laboratory procedures were performed by accredited personnel of the Microbiology and Virology Unit using standardized protocols and validated commercial assays, in accordance with the manufacturers’ instructions. Microbiological and epidemiological data were retrieved from the LIS (DNLAB v.5.3, Dedalus, Milan, Italy) and from the Mercurio v.1.6 epidemiological software (Dedalus, Milan, Italy). The study was conducted in accordance with the Declaration of Helsinki.

### Statistical Analysis

The study population was divided into two groups according to the diagnostic test performed: Group A included patients tested for *Legionella pneumophila* serogroup 1 urinary antigen, and Group B included patients tested for WNV IgG and IgM antibodies.

For each pathogen, the monthly proportion of laboratory-confirmed infections was calculated as the ratio between the number of positive cases and the total number of patients tested in the same month. This proportion was used as a proxy measure of infection burden among clinically suspected patients and as the primary outcome variable for subsequent analyses.

To investigate the association between climatic variables and infection burden, univariate linear regression models were initially applied, considering mean monthly temperature (expressed in °C) and cumulative monthly precipitation (expressed in mm) as independent predictors. These two variables were selected a priori as they were consistently available for the entire study period; average relative humidity, although recognized as a potentially relevant environmental factor, was not included in the analyses because reliable and homogeneous monthly data were not available for the purposes of this study. The significance of regression coefficients was assessed using the *t*-test, while model fit was evaluated using the coefficient of determination (R^2^) and the F-test. Model assumptions were verified by inspection of residuals and by applying the Shapiro–Wilk test to assess normality.

Linear regression models were primarily used as an initial exploratory approach to estimate the direction and strength of associations. Given the potential for non-linear relationships between climatic variables and infection burden (particularly for vector-borne diseases such as WNV), non-linear (GAM) and non-parametric analyses were also applied when appropriate. When outcome variables or residuals deviated from normality, Kendall’s tau correlation coefficients were calculated as a non-parametric measure of association.

Seasonal effects were evaluated using Analysis of Variance (ANOVA), with seasons defined a priori. Homogeneity of variances was assessed using Levene’s test, and when statistically significant differences were detected, Tukey’s post hoc test was applied to identify pairwise seasonal differences.

All statistical analyses were performed using R software version 4.4.2 (R Foundation for Statistical Computing, Vienna, Austria) and Microsoft Excel (Microsoft 2016, Redmond, WA, USA).

## 3. Results

In the study, 10,176 patients were included: 9939 patients in Group A, with an average of 2436 patients/year, and 237 patients in Group B, with an average of 64 patients/year.

The main characteristics of the 97 positive patients for Legionella or WNV are reported for both groups in Table 1. All patients were hospitalized within 3 or 4 days after admission to the emergency department, and none of them were associated with hospital-acquired infection.

For descriptive and analytical purposes, monthly proportions of laboratory-confirmed infections were standardized per 1000 clinically suspected patients tested. Monthly and seasonal averages of temperature and precipitation in Piedmont between January 2021 and September 2024 were also summarized in Table 2.

Summer months (June-August) consistently showed high temperatures with only minor variations, while the winter of 2024 stood out for its unusually elevated mean temperature compared to previous winters. (Table 2).

Regarding precipitation, 2022 was the driest year of the entire study period, recording markedly lower rainfall compared with the surrounding years.

**Figure 1:**
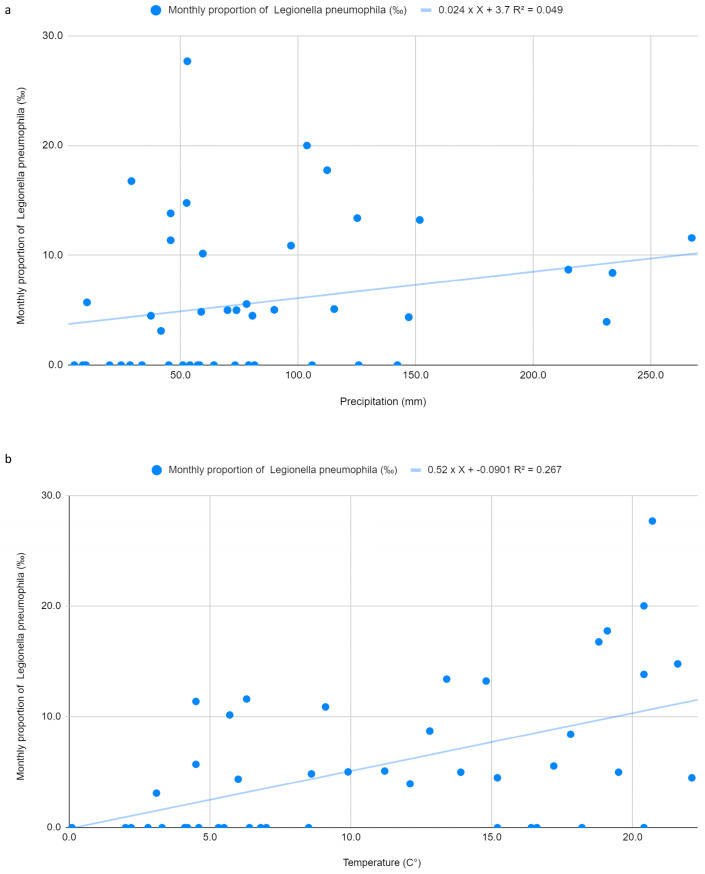
(**a**) Linear regression between monthly proportion of *Legionella pneumophila* serogroup 1 infections (‰) and precipitation; (**b**) Linear regression between monthly proportion of *Legionella pneumophila* serogroup 1 infections (‰) and temperature.

A simple linear regression model assessing the relationship between monthly precipitation (mm) and *Legionella pneumophila* monthly proportions (per 1000 tested patients) did not reveal statistical significance (*p* = 0.1438). Although the estimated coefficient was positive (0.024), suggesting a potential increase in monthly proportions with higher precipitation, the model explained only a small proportion of variability (R^2^ = 0.049) (Figure 1a).

By contrast, average monthly temperature was significantly associated with *Legionella pneumophila* monthly proportions per 1000 tested patients (*p* = 0.000283). The estimated coefficient (0.520) indicated that each 1 °C increase in mean temperature corresponded to an additional 0.52 positive cases per 1000 clinically suspected patients tested. This model showed significant explanatory power for temperature (R^2^ = 0.267) (Figure 1b). Residuals were normally distributed (Shapiro–Wilk *p* = 0.1355), with a standard deviation of 5.814.

A multivariate linear regression model including both precipitation and temperature yielded an R^2^ of 0.284. Precipitation was not statistically significant (*p* = 0.3166), whereas temperature remained highly significant (*p* = 0.000596) with a coefficient consistent with the univariate model (0.496). Residuals again conformed to normality (Shapiro–Wilk *p* = 0.08761). Introducing an interaction term between precipitation and temperature did not meaningfully improve the model (interaction *p* = 0.5296), although the R^2^ increased slightly to 0.291. Since the Shapiro–Wilk test confirmed that the residuals followed a normal distribution were detected, more complex non-linear models were not pursued for *Legionella pneumophila*.

**Figure 2:**
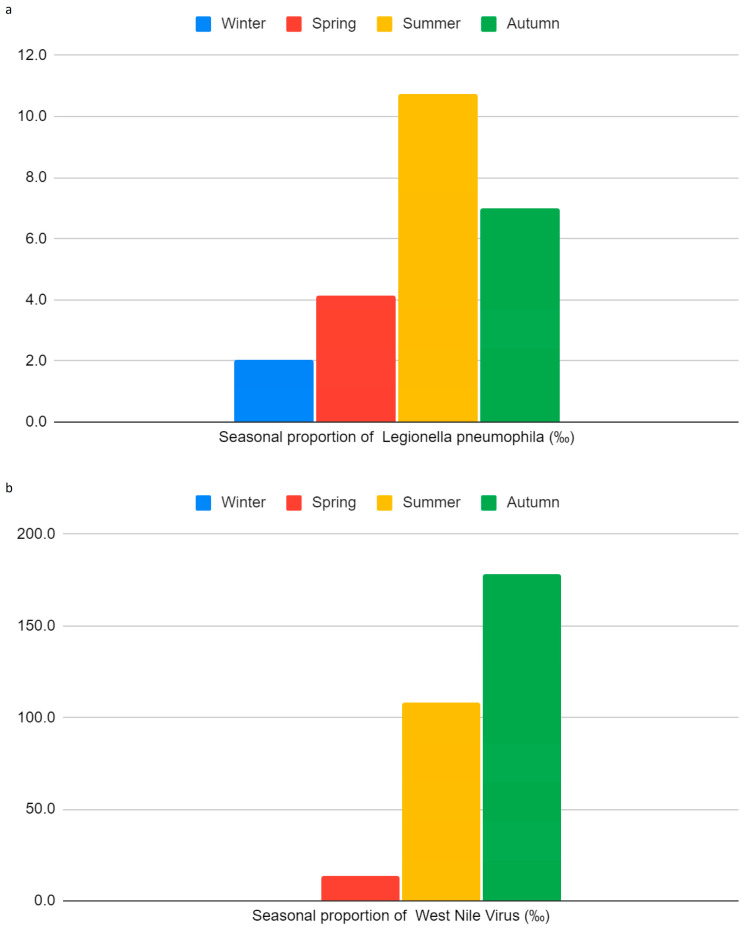
(**a**) Seasonal proportion of *Legionella pneumophila* infections (‰); (**b**) Seasonal proportion of West Nile Virus infections (‰).

Seasonal trends revealed a borderline significant effect on *Legionella pneumophila* monthly proportions (*p* = 0.0598), with the highest values observed in summer (10.72 per 1000 tested patients) (Figure 2a).

**Figure 3:**
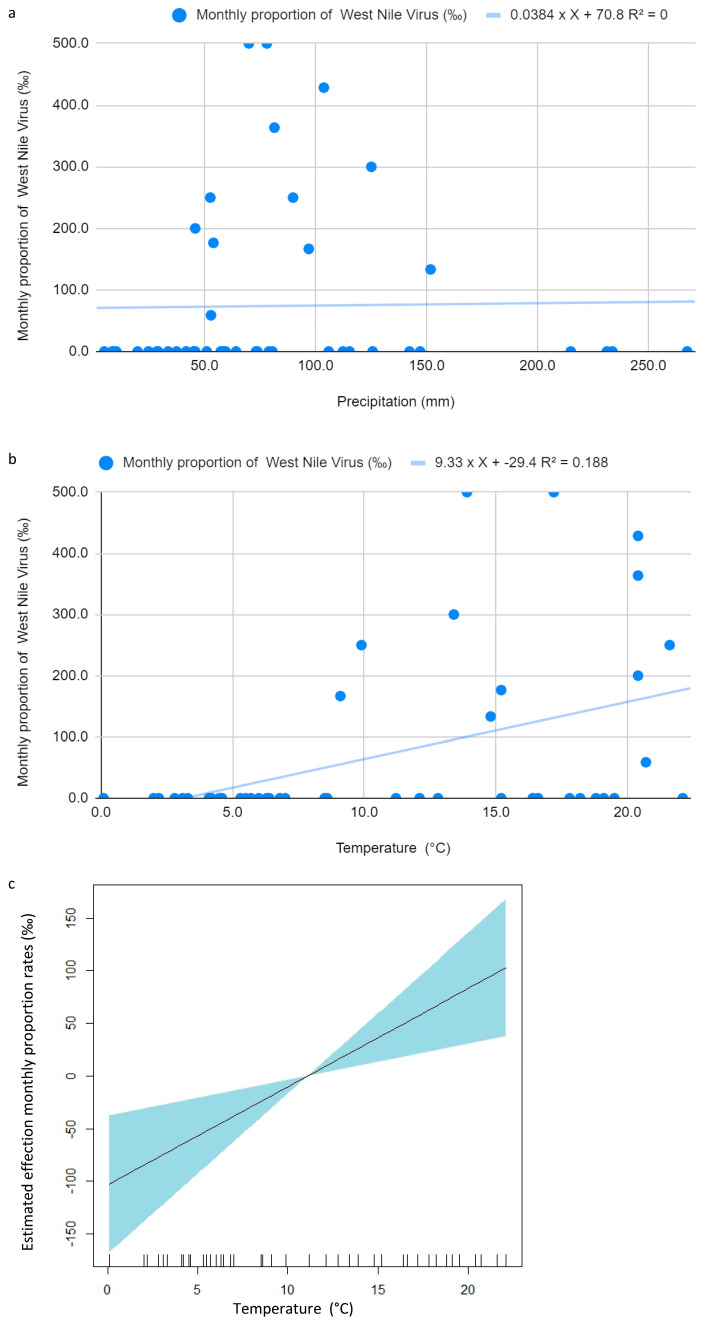
(**a**) Linear regression between monthly proportion of West Nile Virus infections (‰) and precipitation; (**b**) Linear regression between monthly proportion of West Nile Virus infections (‰) and temperature; (**c**) Non-linear relationship obtained using a Generalized Additive Model (GAM) between temperature and monthly proportion of West Nile Virus infections (‰).

For WNV, the linear regression between precipitation and monthly proportions (‰) showed no significant relationship (*p* = 0.914; R^2^ = 0.0003), as illustrated in Figure 3a. However, since residuals deviated significantly from normality (Shapiro–Wilk *p* < 0.00001), a Kendall’s tau-b correlation was performed to ensure a robust non-parametric assessment; this further confirmed the absence of any meaningful association (tau-b = 0.129; *p* = 0.272). In contrast, the mean monthly temperature was a significant predictor of WNV monthly proportions per 1000 tested patients (*p* = 0.00293). The estimated coefficient (9.33) suggested that each 1 °C rise in temperature was associated with 9.33 additional cases per 1000 clinically suspected patients tested (Figure 3b). Although the explanatory power was modest (R^2^ = 0.188), the association remained meaningful.

To address residual non-normality, a Generalized Additive Model (GAM) was applied, confirming the significance of temperature (*p* = 0.00293) and explaining 18.8% of deviance (Figure 3c).

Kendall’s tau correlation also confirmed a significant positive association between temperature and WNV monthly proportions per 1000 tested patients (Tau = 0.3803; *p* = 0.001). WNV seasonality was significant (*p* = 0.00653), with summer again showing the peak monthly proportions (Figure 2b).

## 4. Discussion

The findings of this study reveal a complex and noteworthy relationship between climatic variables and the monthly standardized proportions of *Legionella pneumophila* and WNV infections. Among the environmental factors examined, mean temperature consistently emerged as a stronger determinant of monthly proportions per 1000 patients tested compared to precipitation.

Precipitation appeared to play only a marginal role. Its association with infection burden among clinically suspected patients was not statistically significant for both *Legionella pneumophila* (*p* = 0.1438) and WNV (*p* = 0.914). In contrast, mean temperature showed a statistically significant monthly association. For *Legionella pneumophila*, a significant association was observed (*p* = 0.000283), with a regression coefficient of 0.520. This suggests that each 1 °C rise in average temperature is linked to an increase of 0.52 positive cases per 1000 clinically suspected patients tested. Moreover, the temperature-based model explained a greater share of variability than the precipitation model alone (R^2^ = 0.267).

These results are consistent with recent Italian evidence, which shows that elevated temperatures foster the proliferation of *Legionella pneumophila* in water systems [17]. Of note, in recent years parts of the Piedmont region have recorded an increase of 2–3 °C in both summer and winter, creating favorable conditions for bacterial persistence and transmission.

A multivariate model including both mean temperature and precipitation reinforced the predominant influence of temperature. Although the model’s R^2^ rose modestly (to 0.284), precipitation remained non-significant (*p* = 0.3166), whereas temperature continued to show a significant association (*p* = 0.000596) with a coefficient similar to that of the univariate model (0.496). Introducing an interaction term between the two climatic factors did not meaningfully improve model performance (*p* = 0.5296).

Temperature was also a significant predictor of WNV monthly proportions per 1000 clinically suspected patients tested (*p* = 0.00293), with each 1 °C rise corresponding to an estimated 9.33 additional cases per 1000 clinically suspected patients tested. Seasonal analyses supported these findings: *Legionella pneumophila* monthly proportions per 1000 patients tested peaked in the summer months (mean = 10.72), with a borderline seasonal effect (*p* = 0.0598), while WNV monthly proportions were significantly seasonal (*p* = 0.00653), showing an ‘on-off’ pattern where cases were almost exclusively concentrated in the summer and early autumn (July–October). Indeed, except for a single case in April, no infections were detected during the colder months. This suggests that the relationship with temperature is not strictly linear but is governed by a biological threshold, consistent with the seasonal life cycle of its mosquito vectors; in our setting, WNV circulation became clinically evident only when mean monthly temperatures exceeded 20 °C [1,2,6,19,20]. This threshold effect also explains the non-normality of residuals observed in our linear regression models.

In summary, mean temperature emerged as a major driver of both *Legionella pneumophila* and WNV monthly proportions per 1000 clinically suspected patients tested, whereas precipitation played a limited or negligible role. These insights are particularly relevant in the context of climate change, where rising global temperatures may intensify the burden of climate-sensitive infectious diseases, especially during warmer months.

The main study limitation is related to the single-center, retrospective design that may restrict the generalizability of findings. The focus on symptomatic patients seeking hospital care means that our findings reflect clinically relevant cases (the ‘tip of the iceberg’) rather than the overall prevalence of infection, which includes asymptomatic or mild cases managed in primary care. On the other hand, in a context of climate instability, analyses of local phenomena are advocated to predict regional epidemiological trends more precisely. Secondly, due to the epidemiological nature of the study, both the *Legionella pneumophila* and WNV infection definitions were far from the gold standard. For example, reliance on serological testing for WNV may reduce the temporal precision of infection detection compared to molecular assays such as RT-PCR, potentially weakening associations with climatic variables. In addition, *Legionella pneumophila* monthly proportions per 1000 clinically suspected patients tested were based only on *Legionella pneumophila* serogroup 1 detection. This methodological limitation could underestimate the true burden of disease by overlooking infections caused by other *Legionella pneumophila* serogroups or species. However, this bias could be mitigated by the fact that *Legionella pneumophila* serogroup 1 is the most relevant species and serogroup in our nation.

Another limitation of the present study is the lack of relative humidity data, which may act as an independent climatic driver, especially for *Legionella pneumophila*. The absence of homogeneous monthly humidity measurements may have prevented the identification of additional or synergistic environmental effects. Future multicenter or population-based studies incorporating humidity and other microclimatic variables are warranted.

Finally, although regression-based and non-parametric approaches allowed the identification of robust associations between temperature and infection burden, they do not explicitly account for temporal autocorrelation or delayed climatic effects. Time-series methods, such as autoregressive integrated moving average (ARIMA) models or dynamic regression models, may provide additional insights by modeling lagged relationships between climatic drivers and infection monthly proportions. In particular, delayed effects of temperature increases are biologically plausible for both *Legionella pneumophila* (due to persistence and amplification within water systems) and WNV (where mosquito population dynamics may introduce a delay of several weeks to months between favorable climatic conditions and human infections). Visual inspection of seasonal patterns suggests a possible one-season (approximately 3–4 months) delay between rising temperatures and peak infection proportions. However, the relatively short duration of the study period and the limited number of WNV-positive cases precluded the reliable application of ARIMA-based or dynamic regression models in the present analysis. Future multicenter or population-based studies with longer time series and higher temporal resolution should consider these approaches to better characterize lagged and cumulative climatic effects.

## 5. Conclusions

Our results highlight the critical role of rising temperatures in shaping the seasonal standardized monthly proportions of both *Legionella pneumophila* and WNV among clinically suspected patients. These findings align with growing international evidence linking climate change to the resurgence of infectious diseases once considered rare or well controlled [1,2,4,5,18]. They underscore the urgent need for public health systems to adapt to the evolving risks posed by climate change, with implications not only for health policy but also for broader social, environmental, and economic domains.

## Figures and Tables

**Table 1:** Demographic and Clinical Characteristics of Groups A and B.

Patients’ Features	Group A (n: 57) *Legionella pneumophila*	Group B (n: 40) WNV
Female gender n (%)	10 (18%)	12 (30%)
Mean age, years (min–max)	68 (46–98)	50 (5–87)
Positive cases per month	n (%)	n (%)
January	1 (2%)	0
February	2 (4%)	0
March	3 (5%)	0
April	4 (7%)	1 (3%)
May	5 (9%)	0
June	3 (5%)	0
July	15 (26%)	4 (23%)
August	10 (18%)	11 (28%)
September	4 (7%)	17 (43%)
October	5 (9%)	7 (18%)
November	2 (4%)	0
December	3 (6%)	0
Positive cases per year	n (%)	n (%)
2021	8 (15%)	1 (3%)
2022	5 (9%)	11 (28%)
2023	21 (37%)	17 (43%)
2024	23 (40%)	11 (28%)

**Table 2:** Monthly and seasonal averages of temperature and precipitation in Piedmont between January 2021 and September 2024.

	2021		2022		2023		2024	
Month	Temperature Average (°C)	Precipitation Average (mm)	Temperature Average (°C)	Precipitation Average (mm)	Temperature Average (°C)	Precipitation Average (mm)	Temperature Average (°C)	Precipitation Average (mm)
January	0.1	106	3.3	4.8	2	24.7	3.1	41.7
February	4.1	33.6	4.5	10.2	4.2	9.7	6	147.1
March	5.5	8.4	4.6	19.8	6.8	45	6.3	267.5
April	7	64.2	8.5	58.1	8.6	58.8	9.1	97
May	11.2	115.4	15.2	80.6	12.8	215	12.1	231.3
June	18.2	73.2	19.5	73.8	17.8	233.8	16.6	125.8
July	19.1	112.4	22.1	37.4	20.4	103.8	20.7	52.9
August	18.8	29.1	20.4	81.5	20.4	45.8	21.6	52.6
September	16.4	57.3	15.2	54	17.2	78.1	14.8	151.8
October	9.9	89.9	13.9	70	13.4	125.2		
November	5.3	142.3	6.4	51	5.7	59.5		
December	2.8	28.5	2.2	79	4.5	45.8		
**Season**								
Winter	2.1	69.8	3.5	14.5	2.8	37.8	4.5	78.2
Spring	7.9	62.7	9.4	52.8	9.4	106.3	9.2	198.6
Summer	18.7	71.6	20.7	64.2	19.5	127.8	19.6	77.1
Autumn	10.53	96.5	11.8	58.3	12.1	87.6	7.4	75.9

## Data Availability

The data presented in this study are available within the article and its Supplementary Materials and other data are available on request from the corresponding author.

## Supplementary Materials

The following supporting information can be downloaded at: https://www.mdpi.com/article/10.3390/idr18010018/s1, Table S1: Monthly proportion of laboratory-confirmed Le-gionella pneumophila and WNV infections and monthly averages of temperature and precipita-tions in Piedmont between January 2021 and September 2024.; Table S2: Monthly breakdown by year of positive, negative, and pending samples for *Legionella pneumophila* and West Nile Virus.
