# Supplementary material for: Sickened by the Weather: Exploring the Climatic Impact on West Nile Virus (WNV) and Legionella pneumophila in Piedmont—A Retrospective Observational Study (2021–2024)
Source: Infect Dis Rep. 2026 Feb 12;18(1):18. doi: 10.3390/idr18010018 (PMC12921765; doi:10.3390/idr18010018)
Supplement: Supplementary file 1 [file idr-18-00018-s001.zip › idr-3953402-supplementary.pdf]

Supplement files:

Table S1. monthly proportion of laboratory-confirmed Legionella pneumophila and WNV infections and monthly averages of temperature and precipitations in Piedmont between January 2021 and September 2024.

	**Monthly proportion of Legionella pneumophila (‰)**	**Monthly proportion of West Nile Virus (‰)**	**Rainfall average (mm)**	**Temperature average (°C)**
January 2021	0.0	0.0	106.0	0.1
February 2021	0.0	0.0	33.6	4.1
March 2021	0.0	0.0	8.4	5.5
April 2021	0.0	0.0	64.2	7.0
May 2021	5.1	0.0	115.4	11.2
June 2021	0.0	0.0	73.2	18.2
July 2021	17.7	0.0	112.4	19.1
August 2021	16.8	0.0	29.1	18.8
September 2021	0.0	0.0	57.3	16.4
October 2021	5.0	250.0	89.9	9.9
November 2021	0.0	0.0	142.3	5.3
Dicember 2021	0.0	0.0	28.5	2.8
January 2022	0.0	0.0	4.8	3.3
February 2022	5.7	0.0	10.2	4.5
March 2022	0.0	0.0	19.8	4.6
April 2022	0.0	0.0	58.1	8.5
May 2022	4.5	0.0	80.6	15.2
June 2022	5.0	0.0	73.8	19.5
July 2022	4.5	0.0	37.4	22.1
August 2022	0.0	363.6	81.5	20.4
September 2022	0.0	176.4	54.0	15.2
October 2022	5.0	500.0	70.0	13.9
November 2022	0.0	0.0	51.0	6.4
Dicember 2022	0.0	0.0	79.0	2.2
January 2023	0.0	0.0	24.7	2.0
February 2023	0.0	0.0	9.7	4.2
March 2023	0.0	0.0	45.0	6.8
April 2023	4.8	0.0	58.8	8.6
May 2023	8.7	0.0	215.0	12.8
June 2023	8.4	0.0	233.8	17.8
July 2023	20.0	428.6	103.8	20.4
August 2023	13.8	200.0	45.8	20.4

September 2023	5.6	500.0	78.1	17.2
October 2023	13.3	300.0	125.2	13.4
November 2023	10.1	0.0	59.5	5.7
Dicember 2023	11.4	0.0	45.8	4.5
January 2024	3.1	0.0	41.7	3.1
February 2024	4.4	0.0	147.1	6.0
March 2024	11.6	0.0	267.5	6.3
April 2024	10.9	166.7	97.0	9.1
May 2024	3.9	0.0	231.3	12.1
June 2024	0.0	0.0	125.8	16.6
July 2024	27.5	58.8	52.9	20.7
August 2024	14.8	250.0	52.6	21.6
September 2024	13.2	133.3	151.8	14.8

Table S2. Monthly breakdown by year of positive, negative, and pending samples for Legionella pneumophila and West Nile Virus.

Date	Legionella pneumophila				West Nile Virus			
	Positive	Not received	Negative	Total	Positive	Not received	Negative	Total
January 2021	0	2	204	206	0	0	0	0
February 2021	0	0	195	195	0	0	1	1
March 2021	0	0	259	259	0	0	0	0
April 2021	0	0	241	241	0	0	1	1
May 2021	1	1	195	197	0	0	3	3
June 2021	0	0	169	169	0	0	0	0
July 2021	3	3	166	172	0	0	3	3
August 2021	3	3	176	182	0	0	4	4
September 2021	0	1	152	153	0	0	10	10
October 2021	1	0	198	199	1	2	3	4
November 2021	0	1	237	238	0	0	0	0
December 2021	0	0	205	205	0	0	1	1
January 2022	0	0	192	192	0	0	0	0
February 2022	1	1	174	176	0	0	0	0
March 2022	0	1	191	192	0	0	0	0
April 2022	0	0	191	191	0	0	0	0
May 2022	1	1	221	223	0	0	0	0
June 2022	1	2	199	202	0	0	6	6
July 2022	1	0	221	222	0	0	2	2
August 2022	0	0	208	208	4	0	7	11
September 2022	0	1	183	184	3	0	14	17
October 2022	1	0	199	200	3	0	3	6
November 2022	0	1	190	191	0	0	3	3

Date	*Legionella pneumophila*				West Nile Virus			
	Positive	Not received	Negative	Total	Positive	Not received	Negative	Total
December 2022	0	1	294	295	0	0	2	2
January 2023	0	1	312	313	0	0	3	3
February 2023	0	1	215	216	0	0	0	0
March 2023	0	0	244	244	0	0	4	4
April 2023	1	2	205	208	0	0	6	6
May 2023	2	0	228	230	0	0	4	4
June 2023	2	1	236	239	0	0	5	5
July 2023	4	4	196	204	3	0	4	7
August 2023	3	4	214	221	3	0	12	15
September 2023	1	0	179	180	7	0	7	14
October 2023	3	3	221	227	3	0	7	10
November 2023	2	3	195	200	0	1	8	8
December 2023	3	3	261	267	0	1	1	1
January 2024	1	0	322	323	0	0	7	7
February 2024	1	0	228	229	0	0	7	7
March 2024	3	1	256	260	0	2	8	8
April 2024	3	6	273	282	1	0	5	6
May 2024	1	3	252	256	0	0	8	8
June 2024	0	0	235	235	0	0	7	7
July 2024	7	0	246	253	1	1	16	17
August 2024	4	5	267	276	3	0	9	12
September 2024	3	0	224	227	2	1	13	15
